# Histological evaluation of experimental porcine bruises

**DOI:** 10.1016/j.dib.2018.08.134

**Published:** 2018-09-01

**Authors:** Kristiane Barington, Kerstin Skovgaard, Nicole Lind Henriksen, Anne Sofie Boyum Johansen, Henrik Elvang Jensen

**Affiliations:** a Faculty of Health and Medical Sciences, University of Copenhagen, Ridebanevej 3, DK-1870 Frederiksberg C, Denmark; b Department of Biotechnology and Biomedicine, Technical University of Denmark, Kemitorvet, DK-2800 Kongens Lyngby, Denmark

## Abstract

Age estimation is a crucial part of the forensic investigation of bruises in livestock pigs [1], [2], [3]. Currently, age estimations are based on histological evaluation of the lesions in the skin and underlying muscle tissue [2]. However, the intensity of inflammation and tissue damage depends not only on the age of bruises but also on sampling site, anatomical location and the speed, mass and force used to inflict the lesions [1], [4], [5].

Twelve experimental slaughter pigs were anesthetized and on each animal, four blunt traumas were inflicted on the back (area of impact Nos. 1–4). The pigs were euthanized at 2, 5 or 8 h after infliction. Skin and underlying muscle tissue were sampled from the center (B) and both ends of bruises (A, C) and evaluated histologically. Descriptive statistics were performed on the data obtained and presented in figures and tables. Differences (odds ratios) between sampling sites (A, B and C), object used to inflict bruises (plastic tube or iron bar), anatomical location (area of impact Nos. 1–4) and bruise age (2, 5 and 8 h) were evaluated using the GENMOD procedure in SAS Enterprise Guide 7.1 and presented in tables. In addition, the agreements (estimated as Cohen׳s kappa) between two observers evaluating the histological parameters were calculated and presented. Data have been further analyzed and discussed in a recent paper [1]

**Specifications table**

**Table:** 

Subject area	*Pathology*
More specific subject area	*Forensic veterinary pathology*
Type of data	*Tables, Figures*
How data was acquired	*Microscope*
Data format	*Analyzed*
Experimental factors	*Skin and muscle tissue sampled from experimental bruises in pigs with a body weight of 100 kg*
Experimental features	*Tissue samples were immersion-fixed in formalin, processed through ethanol and xylene and embedded in paraffin. Tissue sections (4–5 µm) were cut and stained with hematoxylin and eosin*
Data source location	*Copenhagen, Denmark*
Data accessibility	*Data published as supplementary material in a research article**[1]*

**Value of the data**

• Assessing the age of bruises is a central part of veterinary and human forensic pathology investigations [1], [2], [3], [4], [5].
• This is the first study of experimental bruises inflicted in slaughter pigs with a body weight (BW) of 100 kg [1]. The experimental setup is comparable to veterinary forensic cases concerning bruises in slaughter pigs [2], [3].
• The data from the present study provide a basis for further studies of bruises in slaughter pigs aiming to improve age estimation of bruises.

## 1 Data

**Fig. 1:**
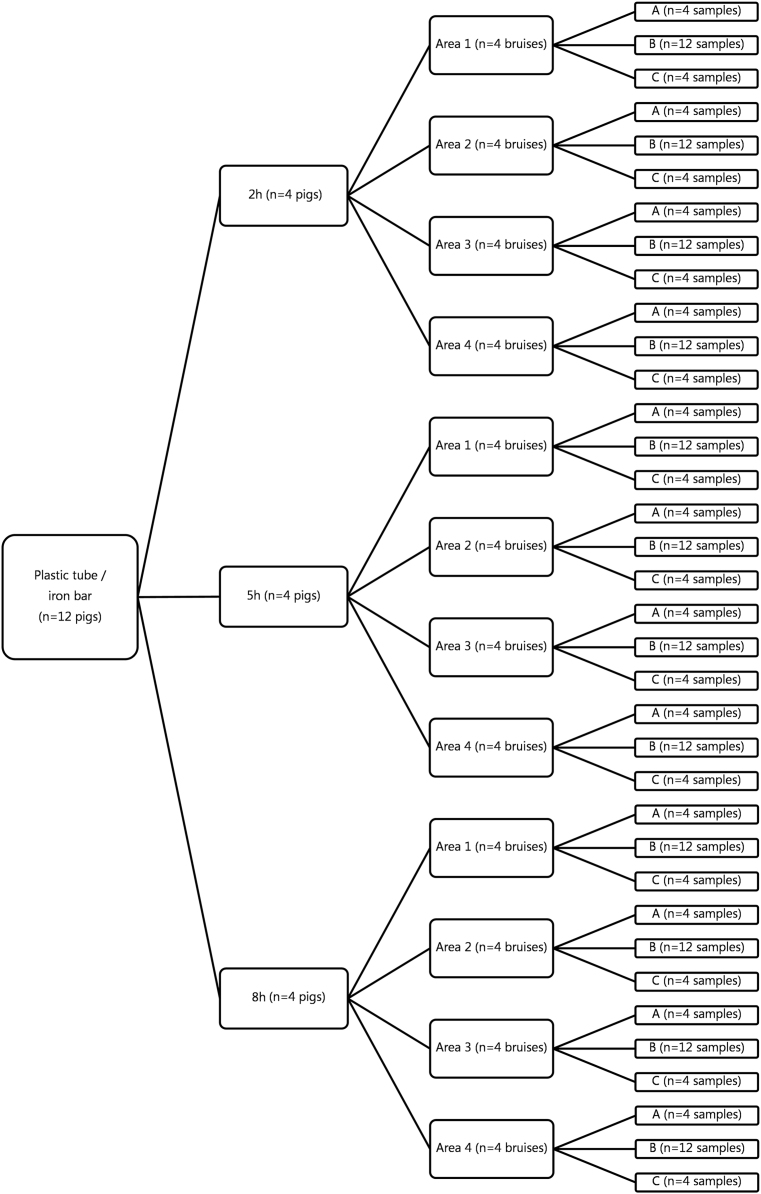
Overview of data. In total, 48 bruises were inflicted on 12 experimental pigs using a plastic tube (*n* = 6 pigs) or an iron bar (*n* = 6 pigs). The bruises were 2, 5 and 8 h old and inflicted on the back of the pigs (area of impact Nos. 1–4). Post-mortem, the skin and underlying muscle tissue were sampled from the center (B) and both ends of the bruises (A, C) and evaluated histologically.

Histological evaluation results of experimental bruises inflicted on pigs weighing in average 100 kg are presented in the present data article. In total, 240 tissue sections from 48 bruises inflicted on 12 pigs were obtained (Fig. 1). Bruises were 2, 5 and 8 h old and inflicted in four areas (area of impact Nos. 1–4) on the back of the pigs (Fig. 1). Bruises were inflicted using either a plastic tube or an iron bar.

Table 1, Table 2, Table 3, Table 4, Table 5, Table 6 present data describing differences in the histological parameters according to the sampling site within a bruise. Table 7 shows the differences in the histological parameters in bruises inflicted either with a plastic tube or an iron bar. Table 8, Table 9, Table 10, Table 11, Table 12, Table 13 present data describing differences in the histological parameters according to the anatomical location of the bruise. Table 14 presents data describing differences in the histological parameters according to the age of bruises. In addition, Table 15 presents the agreement (estimated as Cohen׳s kappa) between two observers carrying out the histological evaluations.

**Table 1:** Histological evaluation of neutrophils and macrophages in the dermis, the subcutaneous fat tissue and underlying muscle tissue from experimental bruises in pigs. Tissue was sampled from the center (B) and both ends (A and C) from a total of 48 bruises. The median, minimum and maximum scores of neutrophils and macrophages are presented according to sampling site. The bruises were between 2 and 8 h of age and inflicted at four anatomical locations.

**Tissue**	**Histological parameter:median (min–max)**	**Sampling site A**	**Sampling site B**	**Sampling site C**	**Control**
Dermis	Neutrophil score	1 (0–3)	2 (0–3)	1 (0–3)	0 (0-0)
Subcutaneous fat tissue	Neutrophil score	1 (0–3)	2 (1–3)	1 (0–3)	0 (0–2)
Subcutaneous fat tissue	Macrophage score	1 (0–2)	1 (0–3)	1 (0–3)	0 (0–3)
Muscle tissue	Neutrophil score	0 (0–3)	0 (0–3)	0 (0–3)	0 (0-0)
Muscle tissue	Macrophage score	0 (0–1)	0 (0–3)	0 (0–3)	0 (0-0)

**Table 2:** Histological absence/presence of hemorrhage (number and percentage of tissue sections) in the dermis underlying the bruises sampled from the center (B) and both ends (A and C).

	**Hemorrhage in the dermis**	
	**Present**	**Absent**
Sampling site A	30 (63%)	18 (37%)
Sampling site B	119 (83%)	25 (17%)
Sampling site C	27 (56%)	21 (44%)
Control	0 (0%)	12 (100%)

**Table 3:** Histological hemorrhage score (number and percentage of tissue sections) in the subcutaneous tissue underlying bruises sampled from the center (B) and both ends (A and C).

	**Hemorrhage in subcutaneous tissue**			
	**0: Absent**	**1: < 12.5%**	**2: 12.5–25%**	**3: > 25%**
Sampling site A	2 (4%)	21 (44%)	19 (39.5%)	6 (12.5%)
Sampling site B	6 (4%)	24 (17%)	43 (30%)	71 (49%)
Sampling site C	11 (23%)	28 (58%)	6 (13%)	3 (6%)
Control	10(83%)	2 (17%)	0 (0%)	0 (0%)

**Table 4:** Histological absence/presence of hemorrhage (number and percentage of tissue sections) in the muscle tissue underlying the bruises sampled from the center (B) and both ends (A and C).

	**Hemorrhage in the muscle tissue**	
	**Present**	**Absent**
Sampling site A	13 (27%)	35 (73%)
Sampling site B	70 (49%)	74 (51%)
Sampling site C	9 (19%)	39 (81%)
Control	0 (0%)	12 (100%)

**Table 5:** Histological muscle necrosis score (number and percentage of tissue sections) in the muscle tissue underlying bruises sampled from the center (B) and both ends (A and C).

	**Necrotic muscle fibers**			
	**0: Absent**	**1: < 12.5%**	**2: 12.5–25%**	**3: > 25%**
Sampling site A	31 (65%)	7 (14.5%)	3 (6%)	7 (14.5%)
Sampling site B	69 (48%)	38 (26%)	8 (6%)	29 (20%)
Sampling site C	34 (71%)	14 (29%)	0 (0%)	0 (0%)
Control	11 (92%)	1 (8%)	0 (0%)	0 (0%)

**Table 6:** Relative differences (odds ratios) between sampling sites A, B and C for the histological parameters in the dermis, subcutaneous tissue and muscle tissue underlaying bruises. Only statistically significant differences (*p*-value < 0.05 and 95% confidence interval (Lower 95 to Upper95) not containing the value 1) are presented.

**Tissue**	**Histological parameter**	**Sampling site**	**Odds ratio**	**Lower95**	**Upper95**	***p*-value**
Dermis	Hemorrhage	B/C	3.8	1.9	8.0	0.0003
Dermis	Hemorrhage	B/A	2.9	1.6	5.1	0.0003
Subcutaneous tissue	Neutrophils	B/C	15.9	5.9	42.8	< 0.0001
Subcutaneous tissue	Neutrophils	A/C	4.8	2.1	10.9	0.0001
Subcutaneous tissue	Neutrophils	B/A	3.3	1.9	5.8	< 0.0001
Subcutaneous tissue	Macrophages	B/C	6.9	2.3	20.7	0.0005
Subcutaneous tissue	Macrophages	A/C	3.9	1.6	9.6	0.0036
Subcutaneous tissue	Hemorrhage	B/C	15.3	5.5	42.3	< 0.0001
Subcutaneous tissue	Hemorrhage	A/C	3.8	2.2	6.7	<0.0001
Subcutaneous tissue	Hemorrhage	B/A	4.0	2.1	7.7	< 0.0001
Muscle tissue	Neutrophils	B/A	4.2	1.9	9.5	0.0005
Muscle tissue	Neutrophils	A/C	2.1	1.0	4.1	0.0394
Muscle tissue	Neutrophils	B/A	2.1	1.2	3.4	0.0048
Muscle tissue	Macrophages	B/C	6.1	2.5	14.9	< 0.0001
Muscle tissue	Macrophages	A/C	3.4	1.6	7.0	0.0012
Muscle tissue	Macrophages	B/A	1.8	1.1	2.9	0.0167
Muscle tissue	Hemorrhage	B/C	4.0	1.4	11.5	0.0105
Muscle tissue	Hemorrhage	B/A	2.5	1.4	4.5	0.0015
Muscle tissue	Necrosis	B/C	3.0	1.6	5.7	0.0005
Muscle tissue	Necrosis	B/A	1.8	1.3	2.6	0.0007

**Table 7:** Relative differences (odds ratios) between bruises inflicted with a plastic tube (P) and an iron bar (I) for the histological variables in the dermis, subcutaneous tissue and muscle tissue underlaying bruises. Only statistically significant differences (*p*-value < 0.05 and 95% confidence interval (Lower 95 to Upper95) not containing the value 1) are presented.

**Tissue**	**Histological parameter**	**Object**	**Odds ratio**	**Lower95**	**Upper95**	***p*-value**
Dermis	Neutrophils	P/I	5.1	1.3	19.2	0.0172
Muscle tissue	Neutrophils	I/P	2.3	1.1	4.6	0.0199
Muscle tissue	Hemorrhage	I/P	5.0	1.7	15.0	0.0042

**Table 8:** Histological evaluation of neutrophils and macrophages in the dermis, the subcutaneous tissue and underlying muscle tissue from experimental bruises in pigs. Tissue was sampled from four anatomical locations (area of impact nos. 1–4) from a total of 48 bruises. The median, minimum and maximum scores of neutrophils and macrophages are presented according to anatomical location. The bruises were 2, 5 and 8 h of age.

**Tissue**	**Histological parameter: Median (min–max)**	**Area**	**Area**	**Area**	**Area**
		**No. 1**	**No. 2**	**No. 3**	**No. 4**
Dermis	Neutrophil score	2 (1–3)	3 (2–3)	3 (1–3)	2 (1–3)
Subcutaneous fat tissue	Neutrophil score	2 (1–3)	2 (2–3)	2 (2–3)	2 (2–3)
Subcutaneous fat tissue	Macrophage score	1.5 (1–3)	1.5 (1–3)	2 (1–3)	2 (1–3)
Muscle tissue	Neutrophil score	0 (0–1)	1 (0–2)	3 (0–3)	3 (1–3)
Muscle tissue	Macrophage score	0 (0–1)	1 (0–2)	2.5 (0–3)	3 (1–3)

**Table 9:** Histological absence/presence of hemorrhage (number and percentage of bruises) in the dermis underlying the bruises inflicted in four areas on the back of pigs.

	**Hemorrhage in the dermis**	
	**Present**	**Absent**
Area No. 1	12 (100%)	0 (0%)
Area No. 2	12 (100%)	0 (0%)
Area No. 3	11 (92%)	1 (8%)
Area No. 4	12 (100)%	0 (0%)

**Table 10:** Histological hemorrhage score (number and percentage of bruises) in the subcutaneous tissue underlying bruises inflicted in four areas on the back of the pigs.

	**Hemorrhage score in subcutaneous tissue**			
	**0: Absent**	**1: < 12.5%**	**2: 12.5–25%**	**3: > 25%**
Area No. 1	0 (0%)	1 (8%)	5 (42%)	6 (50%)
Area No. 2	0 (0%)	1 (8%)	2 (17%)	9 (75%)
Area No. 3	0 (0%)	1 (8%)	0 (0%)	11 (92%)
Area No. 4	0 (0%)	1 (8%)	2 (17%)	9 (75%)

**Table 11:** Histological absence/presence of hemorrhage (number and percentage of bruises) in the muscle tissue underlying the bruises inflicted in four areas on the back of pigs.

	**Hemorrhage in the muscle tissue**	
	**Present**	**Absent**
Area No. 1	4 (33%)	8 (67%)
Area No. 2	9 (75%)	3 (25%)
Area No. 3	10 (83%)	2 (17%)
Area No. 4	12 (100%)	0 (0%)

**Table 12:** Histological muscle necrosis score (number and percentage of bruises) in the muscle tissue underlying bruises inflicted in four areas on the back of the pigs.

	**Necrotic muscle fibers**			
	**0: Absent**	**1: < 12.5%**	**2: 12.5–25%**	**3: > 25%**
Area No. 1	8 (67%)	4 (33%)	0 (0%)	0 (0%)
Area No. 2	1 (8%)	9 (75%)	1 (8%)	1 (8%)
Area No. 3	1 (8%)	1 (8%)	1 (8%)	9 (75%)
Area No. 4	0 (0%)	1 (8%)	0 (0%)	11 (92%)

**Table 13:** Relative differences (odds ratios) between anatomical locations (area of impact Nos. 1–4) for the histological variables in the dermis, subcutaneous tissue and muscle tissue underlaying bruises. Only statistically significant differences (*p*-value < 0.05 and 95% confidence interval (Lower 95 to Upper95) not containing the value 1) are presented.

**Tissue**	**Histological parameter**	**Area No.**	**Odds ratio**	**Lower95**	**Upper95**	***p*-value**
Dermis	Neutrophil score	2/1	4.3	1.6	11.7	0.0037
Dermis	Neutrophil score	2/4	9.2	1.8	47.5	0.0078
Dermis	Neutrophil score	3/4	8.1	1.8	37.1	0.0074
Subcutaneous tissue	Hemorrhage	3/1	8.7	1.1	69.5	0.0420
Muscle tissue	Neutrophil score	2/1	7.9	2.0	31.9	0.0036
Muscle tissue	Neutrophil score	3/1	120.6	13.0	1122.0	< 0.0001
Muscle tissue	Neutrophil score	4/1	501.2	22.5	11,160.9	< 0.0001
Muscle tissue	Neutrophil score	3/2	15.2	2.0	114.0	0.0081
Muscle tissue	Neutrophil score	4/2	63.2	3.6	1.104.9	0.0045
Muscle tissue	Macrophage score	2/1	9.9	1.3	74.1	0.0253
Muscle tissue	Macrophage score	3/1	105.0	11.3	973.6	< 0.0001
Muscle tissue	Macrophage score	4/1	178.4	28.8	1104.3	< 0.0001
Muscle tissue	Macrophage score	3/2	10.6	1.7	67.0	0.0122
Muscle tissue	Macrophage score	4/2	18.0	4.3	74.7	< 0.0001
Muscle tissue	Hemorrhage	3/1	10.0	2.0	50.0	0.0051
Muscle tissue	Hemorrhage	4/1	6.5 × 10^11^	1.3 × 10^11^	3.2 × 10^12^	< 0.0001
Muscle tissue	Hemorrhage	4/2	1.1 × 10^11^	1.9 × 10^10^	6.1 × 10^11^	< 0.0001
Muscle tissue	Necrosis	2/1	10.1	1.7	60.8	0.0116
Muscle tissue	Necrosis	3/1	183.1	15.4	2174.9	< 0.0001
Muscle tissue	Necrosis	4/1	694.4	20.9	23,057.4	0.0003
Muscle tissue	Necrosis	3/2	18.1	2.5	130.9	0.0040
Muscle tissue	Necrosis	4/2	68.8	3.6	1306.3	0.0049

**Table 14:** Histological evaluation of neutrophils and macrophages in the dermis, the subcutaneous tissue and underlying muscle tissue from experimental bruises in pigs. Tissue was sampled from bruises 2, 5 and 8 h old from a total of 48 bruises inflicted at four anatomical locations. The median, minimum and maximum scores of neutrophils and macrophages are presented according to bruise age. The relative difference (odds ratio) in macrophage score in the muscle tissue was significant (*p* =< 0.0001) between bruises 2 h and 5 h old. The odds of bruises 5 h old having a high macrophage score was 2 times the odds of bruises 2 h old having a high macrophage score (95% confidence limits 1.5–2.8). No other relative differences (odds ratios) between bruises 2, 5 and 8 h were found.

**Tissue**	**Histological parameter: Median (min–max)**	**2 h**	**5 h**	**8 h**
Dermis	Neutrophil score	2 (1–3)	2 (1–3)	2.5 (1–3)
Subcutaneous tissue	Neutrophil score	2 (2–3)	2.5 (2–3)	2 (1–3)
Subcutaneous tissue	Macrophage score	1 (1–3)	2 (1–3)	2 (1–3)
Muscle tissue	Neutrophil score	2 (0–3)	2.5 (0–3)	1 (0–3)
Muscle tissue	Macrophage score	1 (0–3)	2 (0–3)	1 (0–3)

**Table 15:** Agreement (estimated as Cohen׳s kappa) between two observers evaluating nine histological parameters in 53 tissue sections of skin and muscle selected randomly from a total of 240 tissue sections from experimental bruises. Limits of 95% confidence interval (Lower95 to Upper95) not including zero and a *p*-value for kappa below 0.05 means that there is some level of agreement between the two observers. The level of agreement was interpreted according to Altman 1991 [6].

**Tissue**	**Parameter**	**Cohen׳s kappa**	**Lower95**	**Upper95**	**Level of agreement**	***p*-value**
Dermis	Neutrophils	0.65	0.51	0.80	Good	< 0.0001
Dermis	Hemorrhage	0.46	0.19	0.73	Moderate	0.0006
Subcutaneous tissue	Neutrophils	0.75	0.63	0.87	Good	< 0.0001
Subcutaneous tissue	Macrophages	0.55	0.37	0.74	Moderate	< 0.0001
Subcutaneous tissue	Hemorrhage	0.82	0.71	0.93	Very good	< 0.0001
Muscle tissue	Necrosis	0.89	0.81	0.97	Very good	< 0.0001
Muscle tissue	Neutrophils	0.90	0.83	0.97	Very good	< 0.0001
Muscle tissue	Macrophages	0.84	0.74	0.93	Very good	< 0.0001
Muscle tissue	Hemorrhage	0.84	0.70	0.99	Very good	< 0.0001

### 1.1 Sampling site

See Table 1, Table 2, Table 3, Table 4, Table 5, Table 6.

### 1.2 Object used to inflict bruises

See Table 7.

### 1.3 Anatomical location

See Table 8, Table 9, Table 10, Table 11, Table 12, Table 13.

### 1.4 Bruise age

See Table 14.

### 1.5 Agreement between observers

See Table 15.

## 2 Experimental design, materials and methods

### 2.1 Experimental setup

The experimental procedure was approved by the Danish Animal Inspectorate (2013-15-2934-00849). A total of 12 pigs were anesthetized using the same protocol as recently described [1], [7]. During a period of 3–4 min, four blunt traumas (area of impact Nos. 1, 2, 3 and 4) were inflicted on the back along the right M. longissimus dorsi from the area just caudal to the scapula and to the lumbar region of each pig using a plastic tube (mass = 0.047 kg, impact speed = 47.4 m/s)or an iron bar (mass = 0.4 kg, impact speed = 19.7 m/s) The blunt traumas were inflicted using a mechanical device and procedure as described recently [1], [7]. All pigs were kept anesthetized during the experiment and 4 pigs were euthanized every 2, 5 and 8 h after infliction of trauma (Fig. 1).

### 2.2 Histology

From each of the areas of impact (Nos. 1–4), 5 slices of skin and underlying muscle tissue were sampled from the center (B, *n* = 3), the dorsal end (A, *n* = 1) and the ventral end (C, *n* = 1) of the bruises. In addition, uninjured skin and muscle tissue were sampled from the right thigh of each pig and served as control tissue. For histology, the samples were fixed in 10% neutral buffered formalin for up to 5 days [8]. Following fixation, tissue samples were processed through graded concentrations of ethanol and xylene [8]. Tissue sections were cut (4–5 µm) and stained with hematoxylin and eosin before all sections (*n* = 240) were blinded and evaluated by a single observer [8]. In addition, 22% of the sections were selected randomly and evaluated by a second observer.

In total, 9 histological parameters were assessed [1]. Neutrophils and macrophages were scored on a semiquantitative scale: (0) Absence of neutrophils or macrophages, respectively; (1) 1–10 neutrophils or macrophages, respectively; (2) 11–30 neutrophils or macrophages, respectively; (3) > 30 neutrophils or macrophages, respectively. The scoring was carried out in the dermis, subcutaneous fat tissue and muscle tissue in a single high power field at 400 fold magnification in the area with the highest density of macrophages and neutrophils. In the dermis and muscle tissue, hemorrhage was registered as present or absent. In the subcutis, the density of hemorrhage was registered as the percentile area of extravasated erythrocytes in a low power field at 100 fold magnification and scored either as (0) absent; (1) minor: < 12.5%; (2) moderate: 12.5–25%; (3) severe > 25%. In the muscle tissue, the percentile area of necrosis was evaluated in the area with the highest density of necrotic muscle fibers and scored according to the following scale in a single low power field at 100 fold magnification: (0) No necrosis: absence of necrotic muscle fibers; (1) minor necrosis: < 12.5%; (2) moderate necrosis: 12.5–50%; (3) severe necrosis: > 50% [1].

### 2.3 Data analysis

The structure of the raw data is presented in Fig. 1.

### 2.4 Sampling site

Bruises of varying age (2, 5 or 8 h) and at anatomical locations (area of impact Nos. 1–4) were pooled. The median, minimum and maximum scores of neutrophils and macrophages in the dermis, subcutaneous tissue and muscle tissue are presented according to sampling site (A, B and C) in Table 1. In addition, data regarding number and percentage of tissue sections with necrotic muscle fibers and hemorrhage in the dermis, subcutaneous tissue and muscle tissue are presented in Table 2, Table 3, Table 4, Table 5.

Differences (odds ratios) according to sampling site (A, B and C) for each of the histological variables were evaluated using the GENMOD procedure in SAS to fit a model to data measured on an ordinal or binary scale and with repeated measures (SAS Enterprise Guide 7.1). Analyzed data are presented in Table 6. The raw data are presented in Supplementary material 2 in Ref. [1].

SAS code for the GENMOD procedure to analyze differences (odds ratios) according to sampling site (A, B and C) for each of the histological variables (outcome):

**proc genmod** data=Sampling_site;

class Pig Samplingsite;

model outcome=Samplingsite/dist=multinomial link=cumlogit;

repeated subject=Pig/corr=ind corrw;

estimate ׳LogORCB׳ Samplingsite **1** -**1**/ exp;

estimate ׳LogORCA׳ Samplingsite **1 0** -**1**/ exp;

estimate ׳LogORBA׳ Samplingsite **0 1** -**1**/ exp;

**run;**

### 2.5 Object used to inflict bruises

Regardless of the sampling site (ends (A and C) or center (B) of the bruise) the maximum scores for each of the histological parameters were registered for each of the bruises. Then bruises of varying age (2, 5 and 8 h) and anatomical location (area of impact Nos. 1–4) were pooled.

Differences (odds ratios) according to anatomical location were evaluated for each of the nine histological variables using the GENMOD procedure in SAS to fit a model to data measured on an ordinal or binary scale and with repeated measures (SAS Enterprise Guide 7.1). Analyzed data are presented in Table 7. The raw data was presented in supplementary file 2 in Ref. [1].

SAS code for the GENMOD procedure to analyze differences (odds ratios) according to object for each of the histological variables (outcome):

**proc genmod** data=Object;

class Pig Material;

model Outcome=Material/dist=bin;

repeated subject=Pig/corr=ind corrw;

estimate ׳LogORPLASTICIRON׳ Material **1** -**1**/ exp;**run**;

### 2.6 Anatomical location

Regardless of the sampling site (ends (A and C) or center (B) of the bruise) the maximum scores for each of the histological parameters were registered for each of the bruises. Then bruises of varying age (2 h, 5 h and 8 h) were pooled. The median, minimum and maximum scores of neutrophils and macrophages in the dermis, subcutaneous tissue and muscle tissue are presented according to anatomical location in Table 8. In addition, data regarding number and percentages of bruises with necrotic muscle fibers and hemorrhage in the dermis, subcutaneous tissue and muscle tissue are presented in Table 9, Table 10, Table 11, Table 12.

Differences (odds ratios) according to anatomical location were evaluated for each of the nine histological variables using the GENMOD procedure in SAS to fit a model to data measured on an ordinal or binary scale and with repeated measures (SAS Enterprise Guide 7.1). Analyzed data are presented in Table 13. The raw data was presented in Supplementary material 3 in Ref. [1].

SAS code for the GENMOD procedure to analyze differences (odds ratios) according to anatomical location (area of impact Nos. 1–4) for each of the histological variables (outcome):

**proc genmod** data=Anatomical_location;

class Pig Anatomicallocation;

model Outcome=Anatomicallocation/dist=multinomial link=cumlogit;

repeated subject=Pig/corr=ind corrw;

estimate ׳LogOR12׳ Anatomicallocation **1** -**1**/ exp;

estimate ׳LogOR13׳ Anatomicallocation **1 0** -**1**/ exp;

estimate ׳LogOR14׳ Anatomicallocation **1 0 0** -**1**/ exp;

estimate ׳LogOR23׳ Anatomicallocation **0 1** -**1**/ exp;

estimate ׳LogOR24׳ Anatomicallocation **0 1 0** -**1**/ exp;

estimate ׳LogOR34׳ Anatomicallocation **0 0 1** -**1**/ exp;

**run;**

### 2.7 Bruise age

Regardless of the sampling site (ends (A and C) or center (B) of the bruise) the maximum scores for each of the histological parameters were registered for each of the bruises. Then bruises inflicted in the four areas of impact were pooled. The median, minimum and maximum scores of neutrophils and macrophages in the dermis, subcutaneous fat tissue and muscle tissue are presented according to bruise age in Table 14.

Differences (odds ratios) according to bruise age were evaluated for each of the nine histological variables using the GENMOD procedure in SAS to fit a model to data measured on an ordinal or binary scale and with repeated measures (SAS Enterprise Guide 7.1). Analyzed data are presented in the legend of Table 14. The raw data was presented in Supplementary material 3 in Ref. [1].

SAS code for the GENMOD procedure to analyze differences (odds ratios) according to bruise age (2, 5 and 8 h) for each of the histological variables (outcome):

**proc genmod** data=Bruise_age;

class Pig Age;.

model Outcome=Age/dist=multinomial link=cumlogit;

repeated subject=Pig/corr=ind corrw;

estimate ׳LogOR2h5h׳ Age **1** -**1**/ exp;

estimate ׳LogOR2h8h׳ Age **1 0** -**1**/ exp;

estimate ׳LogOR5h8h׳ Age **0 1** -**1**/ exp;

**run**;.

### 2.8 Agreement between observers

Agreement between two observers evaluating 53 tissue sections was determined for each of the histological parameters by calculating Cohen׳s kappa or Cohens weighted kappa (SAS Enterprise Guide 7.1). The level of agreement was interpreted according to Altman 1991 [6]. The data are presented in Table 15.
